# Extinction context is learned by pigeons, not given by the environment

**DOI:** 10.1038/s44271-025-00261-2

**Published:** 2025-05-24

**Authors:** Juan Peschken, Lukas Alexander Hahn, Roland Pusch, Jonas Rose

**Affiliations:** 1 Neural Basis of Learning, Institute of Cognitive Neuroscience, Faculty of Psychology, Ruhr University Bochum, Bochum, Germany; 2 Biopsychology, Institute of Cognitive Neuroscience, Faculty of Psychology, Ruhr University Bochum, Bochum, Germany

**Keywords:** Psychology, Animal behaviour, Learning and memory

## Abstract

The saying “context is everything” underscores the importance of interpreting things, be they quotes, events, actions, or stimuli, not in isolation but in the light of a bigger picture - their context. This is evident even in fundamental forms of learning such as extinction learning where, in contextual renewal, an extinguished response reoccurs if the context is changed. But what exactly is context? Is context given by stimuli with inherent properties making them context or, what are the circumstances that allow a stimulus to become “contextual”? Even though the answer may seem intuitively trivial, the literature only provides competing and vague definitions. Using a modified ABA paradigm, we assessed how competing stimuli induced contextual renewal during extinction learning in seven pigeons (*Columba livia*). Furthermore, we controlled the timing of these stimuli and found it to be crucial; with the right contiguity, even small local stimuli resulted in the strongest contextual renewal. This result challenges definitions of context as ‘a backdrop where learning occurs’. Instead, we propose that context can be understood mechanistically as a learned stimulus property. Therefore, context truly is everything and anything.

## Abstract (web summary)

Context is critical yet poorly defined. Using extinction learning in pigeons, we show that context is not given but learned. With the right properties, even small cues can trigger renewal, challenging classical views of context as passive background.

## Introduction

The ability to flexibly adapt to changing environmental conditions is crucial for the survival of any organism. This flexibility requires not only learning and storing of new information but also recognizing when previously acquired information is no longer valid and adjusting behavior accordingly. Central to this capacity is extinction learning.

In extinction, a previously learned association is gradually reduced and may disappear if the expected outcome is repeatedly omitted. For example, a spider-phobic patient would undergo exposure therapy to overcome his fear association, systematically exposing himself to situations involving a spider until the fear diminishes. Extinction learning is widely accepted as the mechanism behind exposure therapy^1,2^. But the association is not simply lost or forgotten, if the context changes after extinction, the association reappears in a phenomenon called renewal^3^. Therefore, at least one component of extinction must be learning to inhibit behavior in a context-dependent manner. The renewal effect has been reported in numerous studies including aversive and appetitive conditioning and in various species including humans, rodents, and pigeons^2,4,5^. Most often, renewal is studied in ‘ABA’ procedures, where each letter denotes a context. An animal first undergoes classical or operant conditioning in context A, it then undergoes extinction in a novel context B, after which it is returned to the original context A where the return of conditioned responses (renewal) can be tested. Renewal can also be observed, albeit to a slightly lesser degree, if the animal is returned to a novel context following extinction in ABC or AAB procedures^6,7^. Most experiments with rodents use different operant chambers or experimental rooms as context. These are commonly enriched with various cues (visual, olfactory, haptic, auditory, spatial) to better capture the idea of a context^6,8^. But renewal has also been reported using interoceptive states and even previous experiences^9,10^. So, what exactly is context?

What constitutes context is the subject of an ongoing debate that has not yet resulted in a unified definition^11^. Central ideas are that context spans long intervals between stimulus presentations^6,12,13^, it arises from “stimuli that comprise the background in which learning occurs” (14, p.248) or is the “set of circumstances around an event” (15, p. 418). In these definitions, context is: centered around physical properties, generally portrayed as multisensorial, continuous, and, therefore, distinct from discrete rapidly changing and highly contingent, learned cues^14–16^. However, recent computational models provide a different perspective on the context in extinction learning. In those models’ contextual stimuli were implemented analogous to cue stimuli without additional a priori assumptions. The models showed context dependent behavior driven solely by associative strength^5,17^. This resulted in a definition of context based only on associative learning properties challenging the distinction between cue and context.

To test these different ideas, we devised a version of the established ABA extinction procedure that introduced competition between different context stimuli. By comparing the strength of renewal between these competing stimuli we were able to determine which acted as the stronger context. We rationalized that if a priori physical stimulus properties determined the context, then environmental information should be an ideal stimulus and would therefore cause the strongest renewal. If, however, associative strength was the decisive factor then contiguity (spatio-temporal simultaneity)^18^ should be critical in making a stimulus the context.

## Methods

### Subjects

We tested seven experimentally naïve pigeons (*Columba livia*), four females and three males, of unknown age. The animals were obtained from local breeders and housed in individual cages within a colony room. During the experimental procedures, all birds had ad libitum access to water and were kept on a controlled feeding protocol, they earned food reward during experimental sessions; if necessary, food was supplemented after the sessions. All procedures were in accordance with the German guidelines for the care and use of animals in science, with the European Communities Council Directive 86/609/EEC concerning the care and use of animals for experimental purposes and approved by the ethics committee of the State of North Rhine Westphalia, Germany.

### Experimental setup

**Fig. 1:**
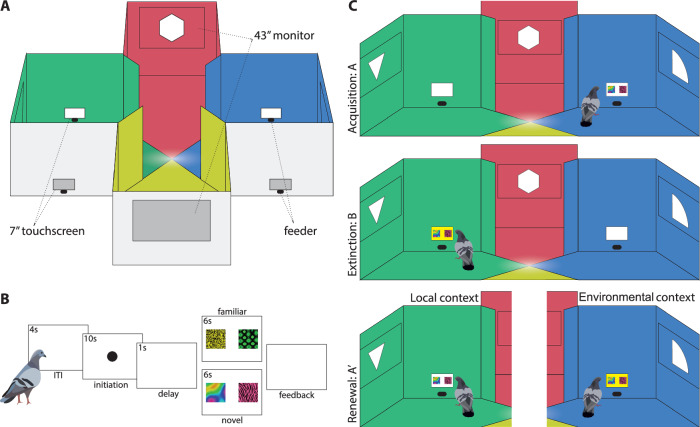
Experimental setup. **A** Arena. Each arm was illuminated in a distinct color and a large monitor displayed a geometrical shape as landmarks. The animals responded on small touchscreens close to the floor, there they also received rewards from automated feeders. **B** Trial structure. Following a peck at the initiation stimulus, a forced choice of S+ and S- was presented. These stimuli were either familiar (stable across all sessions) or novel (session unique) stimuli. Screen-location and sequence of familiar and novel stimuli were pseudo-randomized. **C** ABA’ procedure. Acquisition (context A) took place in a randomly assigned location (balanced across animals and sessions), with the touchscreen showing a white background. Extinction (context B) took place at a different location, with the touchscreen color changed to a distinct color (yellow in the example). Renewal (context A’) was tested either at the acquisition location with the extinction touchscreen color (environmental context) or at the extinction location with the acquisition touchscreen color (local context). A video illustrating the experimental setup and the pigeons’ behavior during the task is available in the supplementary information.

Training and experimental sessions took place in a plus-shaped arena (3.5 m x 3.5 m, Fig. 1A), composed of four identical arms. Programmable LED lamps (Rollei GmbH & Co. KG, Norderstedt, Germany) were mounted above each arm to illuminate it in a unique color. At the end of each arm was a 43” monitor (Dell Inc., Round Rock, United States) that displayed a large stimulus. On both sides of each arm, close to the floor, were 7” touchscreens (Pollin Electronic Ltd., Pförring, Germany), connected to individual Raspberry Pi (version 3 Model B, Raspberry Pi Foundation, Cambridge, United Kingdom). Each Raspberry Pi controlled a mechanical pellet feeder (custom design, delivering Dustless precision pellet 45 mg, Pigeon. Bio-Serv., Flemington, United States), and an illuminated feeding trough. At the center of the arena’s ceiling, a camera was installed to observe and record the birds’ behavior. Additionally, a water bowl was always present at floor level in the center of the arena.

This layout resulted in eight distinctive locations (two per arm) where the birds could interact with a touchscreen and receive rewards, with only one location active at a time. Furthermore, the open design of the setup encouraged more ethologically plausible behavior, as the pigeons moved freely throughout their time in the arena, including flying in and out, resembling their typical foraging behavior.

### Contextual dimensions in the arena setup

The arena constituted a physical setup that allowed controlling context with two distinct stimuli. One was the environmental context, defined by the physical position of the animal in the arena, enriched by a large landmark displayed at the end of each arm and the color of illumination in the arm. A geometrical stimulus (triangle, hexagon, horizontal bar, quarter circle) on a fixed background color (green, red, yellow, blue) served as landmark and the illumination in each arm matched the background color of these screens (Fig. 1A). The landmark and color in any given arm were stable throughout the experiment. The other stimulus serving as context was the local context, which was the background color of the 7” touchscreen on which cue-stimuli were displayed and selected. The colors followed the RGB color model, overall, six different colors were used, the main three colors and their most extreme combinations: Red (1, 0, 0), Green (0, 1, 0), Blue (0, 0, 1), Yellow (1, 1, 0), Cyan (0, 1, 1) and Magenta (1, 0, 1).

The ability of each stimulus to act as a context was assessed by the number of renewal responses after the animal returned to the respective context following extinction. Stimulus presentation and contextual features were controlled by custom MATLAB code (MathWorks, Natick, MA, USA) using the open toolbox for behavioral research^19^ and the Psychophysics toolbox^20^.

### Behavioral protocol

The pigeons were trained to perform a forced choice discrimination task, adapting a well-established protocol (Fig. 1B)^5,21,22^. We presented two pairs of choice stimuli (S+ and S−) in each session, one familiar and one novel to the session. The familiar pair was pre-trained and remained constant across all sessions, serving as control and to maintain task-engagement across the different phases of a session. Conditioned responses to the familiar stimuli (S+) were always rewarded, allowing us to manage satiety levels and potential fatigue during the session. The other pair, novel stimuli, was session unique and had never been presented before. The novel stimuli were the critical stimuli on which acquisition, extinction and renewal were tested. Both familiar and novel stimuli, as well as their location on screen, were presented in a pseudo-randomized and balanced order.

The session began with the pigeon entering freely into the arena, searching for the active touchscreen to initiate the task. A trial started with the presentation of an initiation stimulus; a black dot centered on the touchscreen (for a maximum of ten seconds). The trial was initiated with a single peck to this stimulus, and after a delay (one second), the choice stimuli appeared on screen (for a maximum of six seconds). Subsequently, one of three possible interactions may occur. A peck on the S+ (‘correct response’) resulted in a reward of one food pellet and the illumination of the LED below the feeder for 0.5 s. A peck on the S- (‘alternative response’) resulted in a black screen (one second), no food reward, and an extra delay (one second) before the next trial began. No interaction with either of the stimuli during the presentation period (‘no response’) resulted in no food reward or additional feedback. Consecutive trials were separated by an inter-trial-interval (ITI) of four seconds. Once all trials at a specific location were completed, a black-and-white checkerboard was displayed, signaling the animal that the current touchscreen was no longer active and that it should search for another site.

We adapted the classical three step behavioral protocol ABA^7,23^ to test extinction and renewal. Three distinct phases, acquisition (A), extinction (B), and renewal (A) took place within each session^21^ and were only separated by a one-minute interval. The three phases were accompanied by different combinations of the context stimuli (Fig. 1C).

During acquisition, the subjects learned the corresponding S + /S− association for the novel stimuli by trial and error. The physical array of the arena was constant during each acquisition session (A). Across sessions the environmental context was balanced, so that each of the eight touchscreen-locations was used in only one acquisition. The local context (background color of the touchscreen) was white during all acquisition sessions. Acquisition lasted a minimum of 80 trials (40 for each pair of stimuli) and ended only after the subject achieved 85% correct responses in the last 20 trials for both the familiar and the novel stimuli. Once the acquisition had completed, an ITI of one minute occurred. Here, the bird roamed freely, looking for the next active touchscreen to continue the task in the extinction phase.

Subsequently, the extinction phase took place in a new environment and new local context (B). The animals performed the task at a different touchscreen in a different arm of the arena and the local background changed to a different color. During the extinction phase, responses to the novel stimuli did not result in any feedback (neither food, nor timeout). Pecks on S+ and S- only erased the content of the screen and were followed by the regular ITI. The extinction phase lasted a minimum of 80 trials (40 familiar, 40 novel) and ended only after the subject initiated the minimum number of trials and achieved 85% correct responses on familiar stimuli and 85% extinction on novel stimuli, both measured over the last 20 trials. We defined extinction as initiated trials in which the bird either responded to the S− or omitted a choice response altogether, response to S+ was considered a failure to extinguish. Once extinction had completed, a second ITI of one minute occurred. During this ITI, the animal moved through the arena in search of the final active screen.

Critically, in the renewal phase only one of the two possible contextual stimuli changed back to its acquisition configuration, whereas the other stimulus remained as it was during extinction (renewal phase in context A-prime, A’). This ‘competition’ between the different context stimuli allowed comparing renewal between the different types of contexts. During renewal, the novel stimuli remained without feedback exactly as in extinction. Furthermore, renewal was induced just one minute after the extinction phase had ended. These two factors allowed us to control for potential recovery effects, such as reinstatement and spontaneous recovery, ensuring that the observed responses were a result of the renewal effect. The renewal phase contained a minimum of 40 trials (20 familiar, 20 novel) and ended only after the subject initiated the minimum number of trials and ceased to show renewal responses, achieving 85% of extinction responses in the last 20 trials, (i.e., stopped responding to the unrewarded S+).

### Contiguity manipulation

Experiment I tested the influence of physical dimensions of context stimuli on renewal, Experiment II tested the influence of associative strength. We used two variations of the protocol to test how contiguity (spatio-temporal simultaneity)^18^ of the local context affected the renewal response following the idea that contiguity is one factor determining associative strength.

**Fig. 2:**
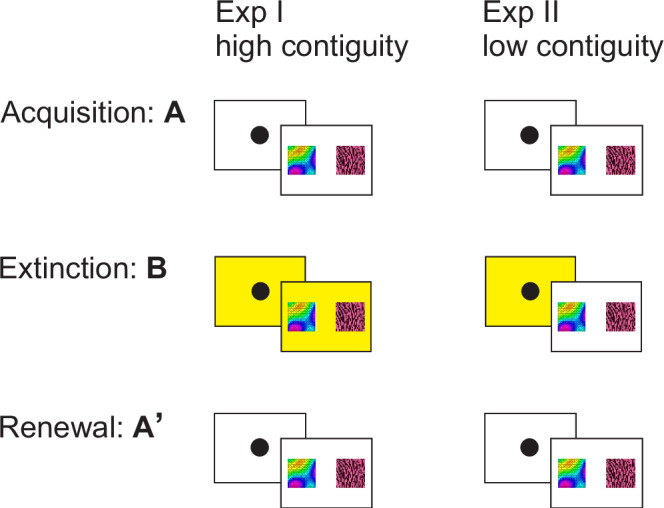
Contiguity manipulation. Experimental manipulations during extinction phase. In Experiment I, local context (background color, yellow in this example) was present on the initiation screen (together with the black dot as initiation stimulus), during the delay and on the discrimination screen (together with the novel S + /S−). In Experiment II the local context was only present on the screen together with the initiation stimulus (about‚ three to four seconds shorter than in Experiment I), i.e., it had lower contiguity than in Experiment I.

In Experiment I, we showed the local context during initiation, delay and choice presentation (Fig. 2, left). Thus, contiguity of the local context to the target S+ and the corresponding feedback was high. In Experiment II we reduced the contiguity of the local context by showing the local context only during presentation of the initiation stimulus, but not during delay and choice presentation (i.e., the touchscreen was white after the initiation, Fig. 2, right). Thus, contiguity of the local context to S+ and feedback was reduced (‘Local –’). This reduction was relatively small as the local context was still presented during active engagement with the touchscreen (initiation) and it reduced exposure to the touchscreen color stimulus only by a short period (the one second delay plus the time it took the animal to make the discrimination decision). Nonetheless, if associative strength were to be the critical component turning a stimulus into a context, we expected to find a weaker renewal effect in Experiment II compared to Experiment I.

The birds were randomly assigned to face a specific context in their first session and then continued with the alternate context in each subsequent session (one session per day on consecutive days), each context was tested four times. Overall, the seven pigeons underwent a total of 112 sessions (16 each), all animals started with Experiment I and finished with Experiment II. Each pigeon had at least 15 days of rest between experiments while they were allowed unrestricted access to food.

### Preregistration

This study was part of an experimental protocol preregistered on the Animal Study Registry platform (animalstudyregistry.org) on January 25th, 2023. Full details of the planned protocol are available at: https://www.animalstudyregistry.org/10.17590/asr.0000305^24^.

The original protocol consisted of three experimental manipulations (E1–E3). This report covers the results of experimental manipulation E2 and E3, named here as Experiment I and Experiment II, respectively. The results of experimental manipulation E1 are stated in a separate report^25^.

We deviated from the original protocol with regard to the statistical analysis. We did not apply t-test and ANOVA statistical tests to our data. We realized that data distribution and the complexity of the dataset, with interacting factors, repeated measures within subjects, and across experimental protocols would not fulfill the assumptions required to perform such testing and would further not result in a comprehensive account of the results. Instead, we decided to perform hypothesis testing with generalized linear mixed models (GLMMs) to allow for an easier and more comprehensive description of the obtained dataset.

### Statistical analysis

The group size in this study was based on a priori power analyses using the software G*Power 3.1^26^. Based on a preliminary pilot study we assumed an effect size of dz = 1.09, a significance level of α = 0.05 and a power of 0.8, resulting in an optimal number of *n* = 7 animals. Custom MATLAB code^27^ was used for general data analysis, GLMM specific analyses were performed using R Statistical Software^28^, using the packages lme4^29^, flexplot^30^ and DHARMa^31^.

To determine successful acquisition, extinction and renewal, we recorded the number of responses and compared them against a pre-defined criterion of 17 out of 20 (i.e., 85%) correct responses in acquisition, and 17 out of 20 (i.e., 85%) extinction responses (i.e., no responses or alternative responses) in extinction and renewal. This criterion is significant (at *p* < 0.05) against random choice at 50%, as described below:

$$P\left(X\ge i\right)=B\left(\!\ge 17|0.5,20\right)={\sum }_{i=17}^{20}\left({n}\atop{i}\right){0.5}^{17}{\left(1-0.5\right)}^{3}=2.0123* {10}^{-4}$$

This equation calculates the probability (*P*) of obtaining at least 17 correct responses out of 20 by chance, assuming a 50% random choice. Using the binomial distribution, the cumulative binomial probability (*B*) of achieving this by chance is 0.00020123 (*p* < 0.05), confirming that reaching the criterion is statistically significant.

We used GLMMs to evaluate the different types of stimuli across phases and to assess how their respective learning properties influenced the number of renewal responses. In the first model, the number of trials to achieve criterion was fit based on three main fixed factors, and one interaction. These factors were experimental phase (Phase; acquisition and extinction), target stimulus (Choice stimulus; familiar and novel) and experimental protocol (Experiment, I and II), the modeled interaction was between Phase and Choice stimulus.

In the second model, number of responses to the novel stimuli was fit based on three main fixed factors with no interactions. These factors were session number (Session; 1, 2, 3, and 4), contextual stimulus (Context; environmental and local) and contiguity (Contiguity; High and Low). We assessed the data distribution and the conformity of the expected residual variance (overdispersion) for both models and determined that a Negative Binomial distribution with a log link function was the most appropriate (refer to the supplementary methods for a complete description). Pigeons were treated as a random grouping effect (bird IDs).

## Results

We observed behavioral responses of seven pigeons in a two-stimulus discrimination task. The animals underwent a within-session acquisition-extinction-renewal paradigm (ABA), in an open arena (see supplementary video file for an overview of the behavioral task). To investigate contextual control of individual stimuli, only one of two context stimuli returned to its acquisition state during the renewal test (ABA’). In Experiment I we implemented two types of context stimuli: an environmental context, and a local context, defined by the background color of the active touchscreen. In Experiment II contiguity of the local context was manipulated by providing the contextual cue solely during the presentation of the initiation stimulus.

**Fig. 3:**
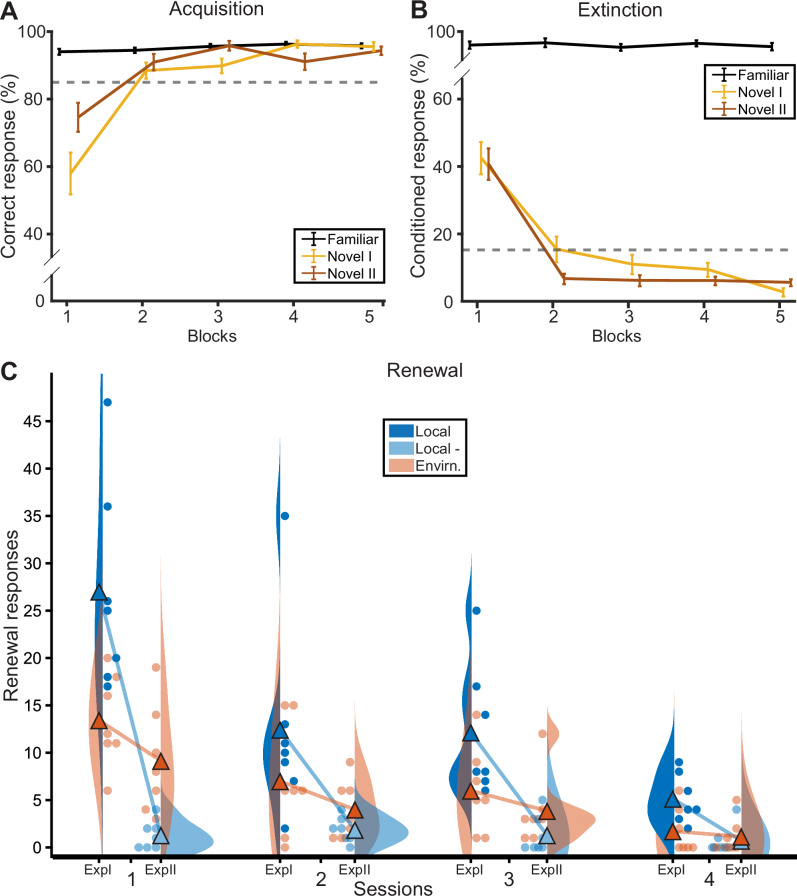
Behavioral results of seven Pigeons. **A** Animals successfully acquired the novel stimuli discrimination in Experiment I and II while retaining high discrimination performance for familiar stimuli (9 trials per block, depicted are mean and standard error across all animals and sessions). **B** Extinction of novel stimuli occurred within the first 20 to 30 trials (i.e., in the second or third block). Rewarded discrimination performance for familiar stimuli stayed high. **C** In Experiment I, both environmental information (Envirn. red) and local stimuli (dark blue) resulted in renewal. Reponses were significantly higher for the local stimuli. In Experiment II the reduction of contiguity of the local stimuli (Local -, light blue) abolished renewal. Repetitions of the extinction protocol reduced the amount of renewal across sessions and experiments. Renewal test started after the end of block 5 in (**B**). Triangles indicate means; dots represent individual data points.

To evaluate the contextual control of behavior across experimental phases, we employed a GLMM to analyze the influence of different types of contexts on the conditioned responses. Pigeons successfully acquired discrimination of the novel stimuli, surpassing the 85% criterion mark for the first time around the 22^nd^ trial (M = 21.65 ± 1.22, Fig. 3A). The birds then underwent extinction of the novel stimuli, following a change of both the environmental and local context. Notably, conditioned responses rapidly declined during extinction (z = 7.641, *p* < 0.001, β = 1.537, 95% CI = [1.146, 1.193]). On average, pigeons achieved the extinction criterion after 26 trials (M = 26.19, ± 1.17). In contrast, the model found no statistically significant evidence that responses to familiar stimuli were affected (z = 1.528, *p* = 0.126, β = 0.330, 95% CI = [−0.092, 0.754], Fig. 3B). Across individuals, performance in both acquisition and extinction exhibited high similarity and low variance (model random effect ${\sigma }^{2}$ = 0.056).

Following extinction of the novel stimuli, we conducted a renewal phase to assess the return of conditioned responses. The influence of the environmental and local context was compared, with only one of the two contextual stimuli reverting to the configuration in the acquisition phase (counterbalanced across animals). We hypothesized that, following common definitions of context, the enriched and continuous environmental stimulus should provide stronger renewal than the small local context stimulus. In Experiment I, we observed robust renewal when reverting either context, (local = 14.17 ± 2.14, environmental = 7.03 ± 1.15), renewal declined across subsequent sessions (Fig. 3C, Table 1). Notably, local context induced more renewal compared to the environmental context (z = 6.743, *p* < 0.001, β = 0.973, 95% CI = [0.692, 1.259]). These effects were consistent across all animals (model random effect ${\sigma }^{2}$ = 0.115).

**Table 1:** Predictor variables on number of renewal responses across both experiments

Coefficients		${{{\rm{Estimate}}}}^{\exp }$	Std. Error	z value	Pr(>|z | )	${95 \% {{\rm{CIs}}}}^{\exp }$	${R}_{{GLMM}}^{2}$
(Intercept)		10.10	0.180	12.782	*p* < 0.001	6.91–14.78	0.728
Contiguity: Low		0.92	0.228	−10.48	*p* < 0.001	0.05–0.14	$\Delta$ −0.486
Session	2	4.88	0.174	−4.180	*p* < 0.001	0.34–0.67	$\Delta$ −0.336
	3	4.53	0.174	−4.595	*p* < 0.001	0.31–0.63	
	4	1.66	0.205	−8.788	*p* < 0.001	0.10–0.24	
Context: Local		26.73	0.144	6.743	*p* < 0.001	1.99–3.52	$\Delta$ −0.182

Within the coefficient ‘Contiguity’ the reference category was ‘High’. For ‘Session’ the reference category was Session 1. For ‘Context’ the reference category was environmental. Estimate and 95% CIs columns have been exponentiated to obtain values in the original scale and facilitate comprehension. The intercept value is the expected number of renewal responses when all predictor variables are at their reference level, values for the predictors were transformed to the original scale and expressed in relation to the intercept (refer to supplementary information for a complete description). ${R}_{{GLMM}}^{2}$ represents the conditional explained variance of the full model, including both fixed and random effects. $\Delta$ quantifies the loss of explained variance when compared with a model without the specific predictor.

In Experiment II, we tested the effect of lowered contiguity of the local context (Fig. 2), while maintaining the environmental context exactly as in Experiment I. We hypothesized that, if renewal was driven by associative learning, i.e., contiguity, then decreased contiguity in the local context should decrease responses. Indeed, decreased contiguity of the local context resulted in complete absence of renewal (z = −10.48, *p* < 0.001, β = −2.391, 95% CI = [−2.849, −1.954], Fig. 3C), indicating that high contiguity of the local cue was essential for it to become the context. In addition, repeated presentations of the environmental context reduced renewal, mirroring the session effect observed in Experiment I (Fig. 3C). Finally, a comparison of reaction times between experiments revealed no significant difference (Experiment I = 1.38, ± 0.19, Experiment II = 1.44, ± 0.27, z = 0.374, *p* = 0.708, β = 0.015, 95% CI = [−0.067, 0.098], refer to the supplementary methods for a complete description).

## Discussion

Here we report an experimental approach, ABA’ extinction, that allows testing renewal as a function of different context stimuli. In Experiment I we tested context stimuli with vastly different physical properties and found that returning to either the environmental context (continuous visuospatial surrounding) or the local context (small local cue) resulted in renewal. Contrary to our expectation, we found that it was the local, not the environmental context, that resulted in the strongest renewal. In Experiment II we proceeded to explore the influence, not of physical stimulus properties, but of the associative strength of context stimuli, on renewal. We manipulated the associative strength of the stronger (local) context stimulus by reducing its contiguity. This manipulation all but abolished renewal when the animals were returned to this (local -) context of acquisition.

Typically, contextual variables are examined in isolation, where only one manipulated stimulus becomes context, or collectively, all contextual stimuli revert to the acquisition state simultaneously. Such testing leads animals to easily perceive a salient difference between acquisition, extinction, and subsequent renewal phases. This method makes it difficult to find a mechanistic definition of context since any salient difference in an animal’s state or surrounding can result in large renewal effects^7^. In contrast, our approach allows for directly testing what makes a stimulus the context by creating competition between available stimuli and in turn manipulating individual context stimuli. When we found that the local context, rather than the environmental context, produced the strongest renewal, we proceeded to determine what properties turn this local cue into a context. The literature suggests that attention mechanisms^32,33^ and learning processes^34,35^ play key roles in context formation. Our contiguity manipulation revealed that the ability of the local stimulus to serve as context critically depends on contiguity.

Consequentially, one might expect that other aspects of learning also influence context formation. The most likely candidates are other classic parameters of associative learning: contingency^36,37^ and salience^38^. While in our current design the contingency of all stimuli is the same, preventing us from drawing any conclusions (i.e., when the stimulus is present, no food is available), this may not be the case for salience.

Saliency is a key factor in associative learning, and if—and only if—our proposal is correct, it should also play a role in the learning of context. If our conclusions were incorrect and context were best understood as merely the environment of the animal, the situation would be quite different. Context would not fully obey learning rules, and the environment would inherently serve as the optimal contextual stimulus. This classical perspective, which defines context as the environment, would not predict a direct relationship between the strength of renewal and saliency, as animals would only need to detect environmental changes. However, our findings contradict this view: pigeons exhibited renewal with the environmental context—indicating it was salient enough to be recognized as context—yet renewal was consistently weaker than with the local context.

A logical prediction would then be that a more salient stimulus is more likely to dominate competition and be learned as the contextual cue, or should in principle, produce greater renewal. In an additional experiment (mentioned in our preregistration), we indirectly tested this prediction using different implementations of the environmental stimuli. Although there are differences between the protocol used in that study and the one reported here, the implementation of the environmental context in that experiment was ‘less salient’. When comparing renewal between this ‘less salient’ environmental context and the current ‘high salience’ one, the prediction holds true: greater saliency leads to stronger renewal^25^. This observation supports our interpretation that any stimulus can be learned as a context, including the visuo-spatial information in the environmental context, with its effectiveness being shaped by learning parameters (such as salience or contiguity) rather than by inherent stimulus properties.

At this point, it is essential to consider alternative explanations, particularly regarding our manipulation and the species used, that could account for the observed response pattern. For instance, the manipulations of the contiguity of the local context could also be expressed as a difference between processing simultaneous and sequential information, this is key since it has been argued that pigeons seem to come more easily under the control of simultaneously than sequentially presented information^39^. This would imply that our animals may have failed to encode the local context and omitted its information. However, the manipulation was very short, reducing only the time interval between the initiation screen and the discrimination screen (by one second). In both classical^40^ and modern studies^41^, it has been demonstrated that pigeons can respond to, and modulate their behavior based on stimuli presented much earlier in time (up to 60 seconds^40^), in addition, their ability to improve both reference and working memory^42^ has been shown.

Another consideration is pigeons’ known sensitivity to local rather than global stimulus features, which aligns with our results^43^. However, this tendency can shift when task parameters are modified, particularly when exposure time to relevant stimuli is extended^44^. In our study, animals had up to six seconds to respond to the choice stimuli, and mean reaction times did not differ significantly between experiments. This suggests that pigeons had sufficient time to process both local and global (environmental) features. Moreover, we found no evidence that their behavior changed between experiments, indicating that the same processing mechanisms applied in both cases. Since Experiment I strongly demonstrated that the local context produced renewal, we infer that in Experiment II, pigeons did sample the local information, but it competed with the now more dominant environmental stimulus. Ultimately, this competition led the local context to ‘lose the battle’ and fail to reliably establish itself as the context.

Our results allow us then to challenge the classical idea of context as a stable (continuous) backdrop against which learning occurs^12–14^ in two ways. We find comparably little renewal with the environmental context, a direct implementation of the classical context definition (multisensorial continuous information surrounding the animal). In contrast, the local context was discrete and discontinuous, with very little temporal exposure, some might even consider it a cue rather than a context. Yet, remarkably, this local context resulted in comparably large renewal.

When blurring the distinction between cue and context, occasion setters come to mind. These are often described as less salient stimuli that precede the target stimulus and are learned more easily due to their diffuse and multisensory nature^45^. In extinction learning, occasion setting is believed to play a role in Pavlovian procedures where occasion setters may modulate associations between conditioned and unconditioned stimuli^46,47^. In operant procedures, however, there is evidence for a more direct inhibitory role of the context on specific responses^48–50^. Regardless of this notion, it is worth pointing out, that the definition of occasion setters closely resembles the classical definition of context that we challenge here, thereby opening the door to revisiting the proposed differences between occasion setting and context in operant extinction.

Our results further challenge the classical idea of context as a stable (continuous) backdrop against which learning occurs by demonstrating that the rules of associative learning determine which stimuli are context. Reducing the contiguity of the local context virtually abolished renewal. This means that stimuli serving as context may not easily be distinguished from other types of cues. This questions the assumption that only certain stimuli possess the physical properties required to serve as context, and that cues and context are fundamentally different in nature^14–16^. By moving beyond such classical definitions of context, we can now define context mechanistically. This mechanistic account can explain why a wide variety of contexts have been reported to work effectively in different setups, many of which produce clear extinction and robust renewal.

Multiple models have been proposed to explain how agents assign relevance to context, which in turn helps to understand renewal in extinction learning. One mechanism involves attentional processes that make information context-dependent^32,33^. Conversely, the formation of latent causes could allow the agent to select among various associations stored in memory between a response and the same stimulus^34,35^. Alternatively, based on past experiences, distinct representations can be formed for each context, forcing the agent to consider the context representation when making decisions^33^.

According to the Attentional Theory of Context Processing (ATCP)^33^, organisms tend to ignore contextual information when it lacks significance—such as when the CS is unambiguous and provides all the necessary information to solve the task. During extinction, however, when the CS becomes ambiguous, organisms shift their attention to the context, leading to context-specific processing. The model suggests that in complex stimulus environments, some stimuli act as cues while others form the background. This distinction depends on factors like contiguity, the contingency between target and outcome, and the relative salience of target versus background stimuli. As a result^33^, proposed an operational definition of context as “all of the background stimuli that are irrelevant to the task and ignored by participants until the task becomes ambiguous” (37, p. 150).

In contrast^35^, propose the latent cause theory of conditioning, which suggests that learning CS-US associations is shaped by the animal’s probabilistic beliefs regarding underlying latent causes. Prediction error causes failure to predict the observed CS-US contingency accurately, forcing the animal to update its beliefs. The transition from acquisition to extinction involves significant changes in sensory input, causing the animal to categorize acquisition and extinction trials as belonging to different latent causes. In this model, context is operationally defined as the “history of sensory data”^35^ (p. 4).

Finally, while^17^ remained agnostic on a definition of context, they demonstrated that a memory-driven agent employing reinforcement learning alongside a deep learning network could create independent representations for different contexts based solely on raw sensory inputs, without explicitly distinguishing cues and contexts. This network would then be compelled to consider the relevant context in each trial to account for context-dependent behavior, such as renewal.

Although our study does not primarily aim to explain the mechanisms behind the context dependent behavior in extinction learning, our paradigm allows us to inform these models by providing a precise account on how stimuli become context. When prediction error leads organisms to attend the context, they do not treat all stimuli equally. Instead, stimuli that are more readily learned through their associative properties become the defining contextual cues.

### Limitations

Although we report observational evidence on the role of stimulus salience^25^, a limitation of the current study is the lack of a direct method to quantify and control the relative salience of contextual cues. As a result, we are limited in our ability to draw definitive conclusions about how salience contributes to context formation (It should be noted, however, that whatever the relative salience may have been, it was sufficient for both stimuli in this study to be sampled; refer to the previous discussion). Future studies would benefit from developing systematic approaches to measure and manipulate salience across stimuli, which would allow for a more precise understanding of its influence on learning and behavioral flexibility.

A second limitation, closely tied to the role of salience, is attention. While our behavioral data offers direct insight into which stimuli and contextual changes influence responses, we currently lack an objective measure of attentional allocation during critical phases of the task. Gaining access to such a measure could substantially deepen our understanding of the learning processes involved.

One promising direction for future research would be to combine behavior with single-unit electrophysiology to examine how attention and stimulus representations evolve during extinction. However, the open and “free-moving” nature of our experimental arena poses challenges for the kind of tightly controlled analyses typically expected in electrophysiological studies. If we aim to obtain an objective readout of attentional allocation, implementing visual gaze tracking would be a logical step, allowing us to determine where in space, and thus to which contextual cues, the animals are attending during extinction and renewal. Nonetheless, significant technical challenges remain, particularly for monitoring the visual gaze in pigeons, whose wide visual fields and unrestricted locomotion complicate the use of conventional eye-tracking methods.

## Conclusions

Revising our understanding of context to reflect an active process governed by associative learning rules provides a robust mechanistic explanation for why a wide range of stimuli can serve as context, while also clarifying the role of context in extinction learning. Additionally, our protocol enables a systematic investigation of the factors that contribute to contextual relevance. Classical learning theory elements—such as contingency, salience, and contiguity—raise key questions that could further enhance our understanding of context as a tool for disambiguating information. These insights could offer valuable parameters for future experimental research and computational models aimed at capturing this complexity, ultimately enabling the search for precise neural correlates and causal manipulations that may lead to a definitive understanding of context.

Together, we demonstrate that context appears to emerge from an active process of disambiguating information in the environment, and we present an approach for testing it in experimental settings. Ultimately, this work lays the groundwork for a deeper understanding of context in extinction learning and a mechanistic definition of context.

## Supplementary information

Peer review file

Supplementary Information

Description of additional supplementary video

ABA' paradigm

## Data Availability

The full dataset generated in this study is publicly available on Zenodo at https://zenodo.org/records/15276159^51^.

## Code availability

The custom code used for the analysis in this study is publicly accessible via Zenodo at https://zenodo.org/records/15276159^51^.
