# Supplementary material for: Extinction context is learned by pigeons, not given by the environment
Source: Commun Psychol. 2025 May 24;3:83. doi: 10.1038/s44271-025-00261-2 (PMC12103601; doi:10.1038/s44271-025-00261-2)
Supplement: Supplementary file 2 — Supplementary Information [file 44271_2025_261_MOESM2_ESM.pdf]

Supplementary information

Supplementary Methods

We deviated from the analysis in [1] due to the complexity of the dataset. We realized that data distribution, repeated measures within subjects, multiple experimental protocols, and interacting factors would not be adequately analyzed within a traditional hypothesis-testing framework. Instead, we decided to fit Generalized Linear Mixed Models (GLMMs) to the data, allowing for a more comprehensive and accurate analysis.

The process of constructing the models and subsequent comparisons followed a predefined approach based on [2–4]. First, an inspection was conducted to determine which possible distribution would best fit the data. Initially, the nature of the response variables (trials to criterion and renewal responses), being positive integer values, guided us to consider a Poisson distribution. However, the variance in the different conditions showed a clear deviation from the mean. Further testing revealed that a Negative-Binomial distribution could account for the variance in the data and provided a sound alternative. Next, multiple versions of the models were fitted according to the hypotheses and interpretation of the data. Comparisons were made on the basis of AIC, BIC, and Bayes Factor, resulting in the selection of the final model. Lastly, a validation of the final model was performed based on an analysis of the residuals.

Two different models were fitted to account for the transitions between phases. Model one explored the changes in trials required to achieve the criterion on familiar and novel stimuli in the transition from acquisition to extinction. Here, the variable is standardized to be 0 if the animal took the absolute minimum trials to achieve the criterion (17 trials). Any further number of trials required to achieve the criterion was then an increment to the standardized 0. For example, an animal that required 20 trials to achieve the criterion in a specific session would have a value of 3 for that specific data point. The goal here was to assess how changes in context and reward contingencies affected performance on both stimuli. Based on the paradigm, we had two hypotheses: (i) The familiar stimuli, since they are pre-trained, should not be distinct from 0 and should show very little variation, if any, between phases (contexts). (ii) The novel stimuli, since they have to be learned by trial and error in each session, should show a difference compared to the familiar stimuli.

Indeed, results from model one suggests that the intercept, which represented the value for familiar stimuli during acquisition, was not different from 0 ($z = -1.025$, $p = 0.305$, $\beta = -0.199$, 95% CI = [−0.059, -0.185]). Similarly, the interaction between phase and stimuli ($z = 1.299$, $p = 0.194$, $\beta = 0.351$, 95% CI = [−0.180, -0.881]) and phase as a main factor ($z = 1.528$, $p = 0.126$, $\beta = 0.330$, 95% CI = [−0.092, -0.754]) were not significant. This indicates that the trials to achieve criterion for both stimuli were quite stable across the different phases (contexts).

However, a significant difference between stimuli was found, novel stimuli required more trials to achieve criterion (z = 7.641, p < 0.001, β = 1.537, 95% CI = [1.146, 1.935]). Finally, an additional exploration of the different experiments found a faster achievement of the criterion in Experiment II compared to Experiment I (z = -4.542, p < 0.001, β = -0.597, 95% CI = [-0.856, -0.339), indicating a possible learning effect due to the repetition of the protocol. Resulting in an overall fewer number of trials to achieve the criterion between the acquisition and extinction phases in the second experiment.

Table SI1. Coefficients of predictor variables on number of trials to criterion across both experiments for acquisition and extinction. Within the coefficient ‘Phase’ the reference category was ‘acquisition-ACQ. For ‘Stimuli the reference category was ‘familiar’. For ‘Experiment’ the reference category was I. Estimate column is presented in the raw model output (log link function for Negative-Binomial).

Coefficients	Estimate	Std. Error	z value	Pr(>|z|)	95% CI
(Intercept)	-0.199	0.194	-1.025	p = 0.305	-0.059 – 0.185
Phase EXT	0.330	0.216	1.528	p = 0.126	-0.092 – 0.754
Stimuli Novel	1.537	0.201	7.641	p < 0.001	1.146 – 1.935
Experiment II	-0.597	0.131	-4.542	p < 0.001	-0.856 – -0.339
Phase EXT: Novel	0.351	0.270	1.299	p = 0.194	-0.180 – 0.881

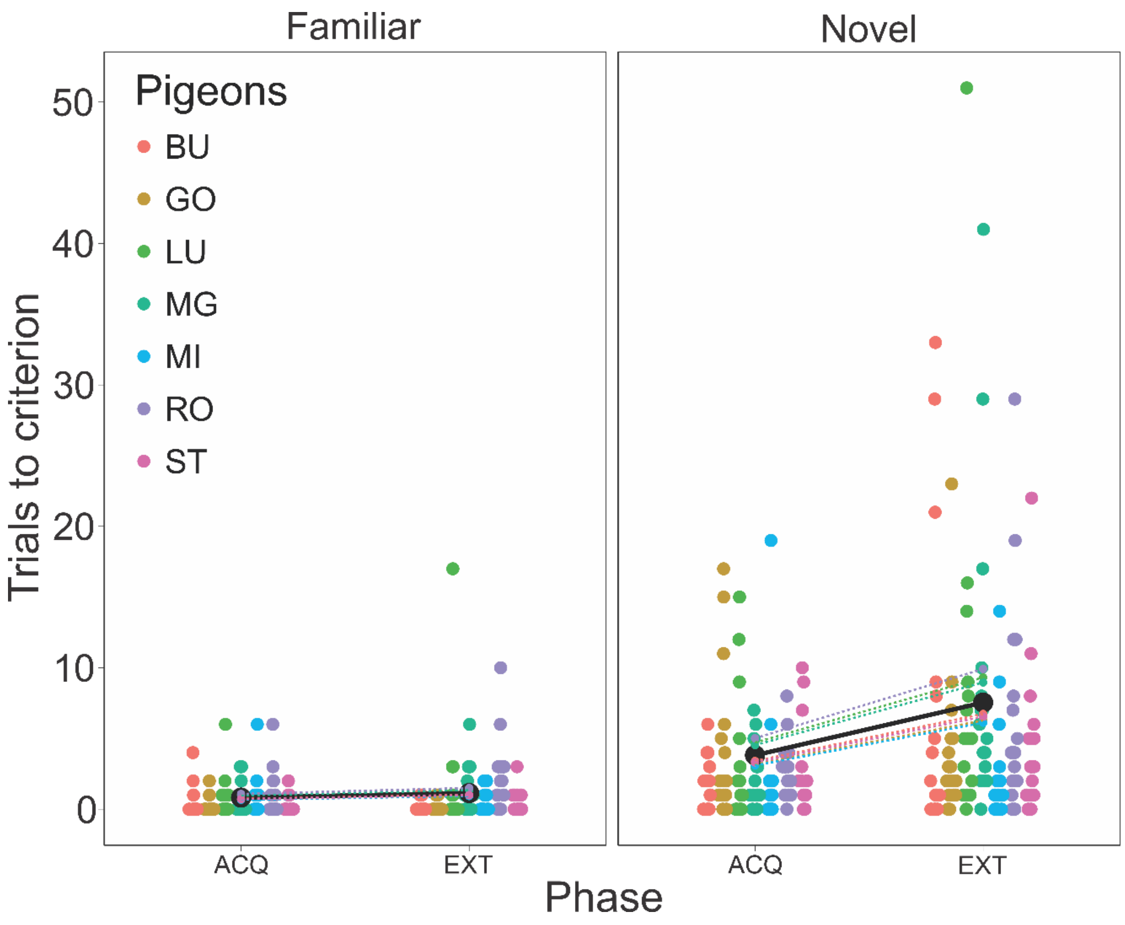

Fig. SI1. Visual representation of model 1. Solid black line represents the main difference across phases, dashed colored lines visualize the individual slope for the random term (Pigeons - each color stands for one specific subject). Dots signalize the real number of trials to reach criterion for each pigeon in each session.

Table SI2. Coefficients of predictor variables on number of renewal responses across both experiments. Within the coefficient 'Contiguity' the reference category was 'High'. For 'Session' the reference category was Session 1. For 'Context' the reference category was Local. Estimate column is presented in the raw model output (log link function for Negative-Binomial).

Coefficients		Estimate	Std. Error	z value	Pr(>|z|)	95% CI
(Intercept)		3.285	0.194	16.87	$p < 0.001$	1.933 – 2.699
Contiguity: Low		-2.391	0.228	-10.48	$p < 0.001$	-2.849 – -1.954
Session	2	-0.727	0.174	-4.180	$p < 0.001$	-1.069 – -0.386
	3	-0.801	0.174	-4.595	$p < 0.001$	-1.144 – -0.460
	4	-1.808	0.205	-8.788	$p < 0.001$	-2.216 – -1.409
Context: Local		0.973	0.144	6.743	$p < 0.001$	0.692 – 1.259

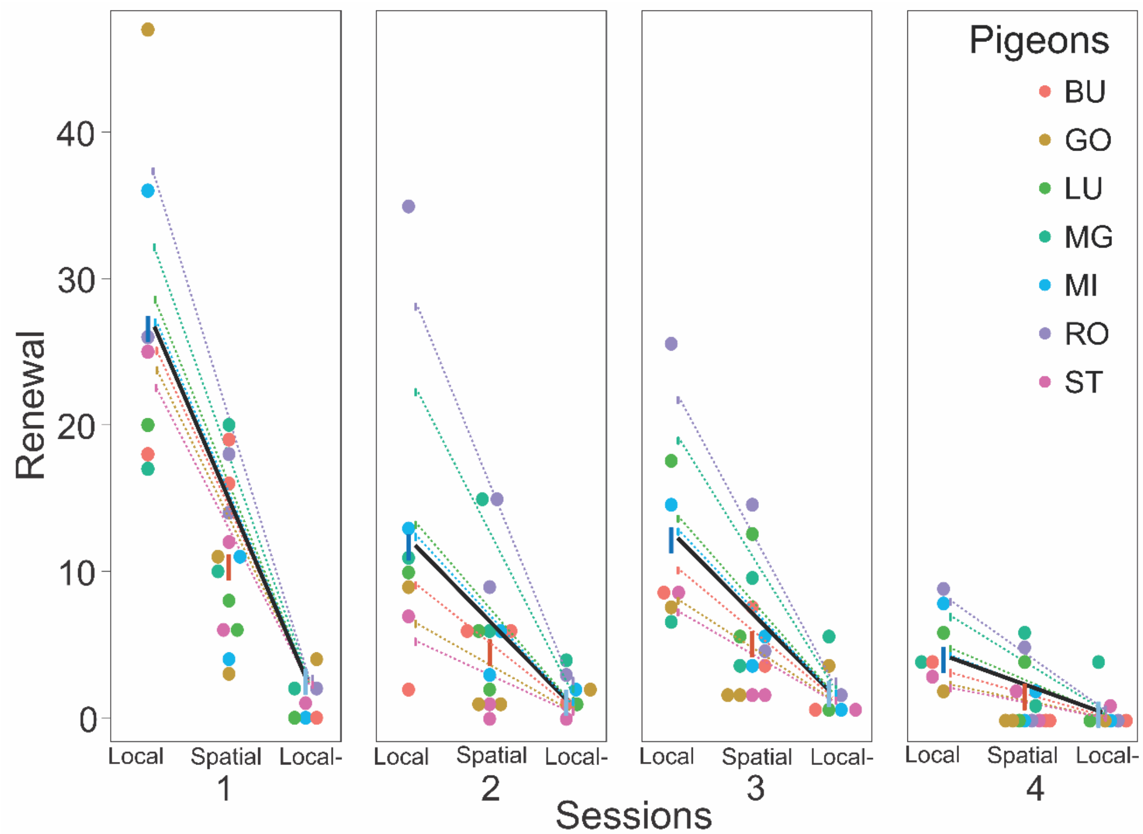

Fig. SI2. Visual representation of model 2. Solid black line represents the main effect of contiguity in the different sessions, dashed colored lines visualize the individual slope for the contiguity effect in the random term (Pigeons - each color stands for one specific subject). Dots signalize the real number of renewal responses for each pigeon. Small colored bars represent the model prediction for each specific subject, thick colored bars represent the model prediction for each context (Local, Local -, environmental here Spatial), color convention follows the scheme presented in Fig. 3C

A third and final model was fitted to compare reaction times (RT) between extinction trials of the novel stimuli during extinction in Experiment I and Experiment II. Since the response variable was time, expressed as a continuous measurement, we employed a gamma distribution with a log link function and replicated the structure of the random grouping effect by bird IDs.

Table SI3. Coefficients of predictor variables on reaction times in extinction trials across both experiments. Within the coefficient 'Experiment' the reference category was 'Experiment' I'. Estimate column is presented in the raw model output (log link function for the Gamma distribucion).

Coefficients	Estimate	Std. Error	z value	Pr(>|z|)	95% CI
(Intercept)	0.318	0.069	4.618	p < 0.001	0.183 – 0.453
Experiment II	0.015	0.042	0.374	p = 0.708	-0.067 – 0.098

Results from the model suggest that the intercept, which represented the reaction time for extinction trials in Experiment I, was different from 0 ($z = 4.618$, $p < 0.001$, $\beta = 0.318$, 95%CI = [0.183, 0.453]). However, the model found no statistically significant difference in reaction times in Experiment II when compared to Experiment I ($z = 0.374$, $p = 0.708$, $\beta = 0.015$, 95%CI = [-0.067, 0.098]). This model was fitted in R Statistical Software [5], using the package glmmTMB [6].

Supplementary references

(1) Medina Peschken, J. J. *Context as a Learned Property of Stimuli in Extinction Learning*; Animal Study Registry, 2023; p 118,560 bytes. https://doi.org/10.17590/ASR.0000305.

(2) Inchausti, P. *Statistical Modelling with r: A Dual Frequentist and Bayesian Approach for Life Scientists*; Oxford University Press: New York, 2022.

(3) Fife, D. The Eight Steps of Data Analysis: A Graphical Framework to Promote Sound Statistical Analysis. *Perspect Psychol Sci* **2020**, *15* (4), 1054–1075. https://doi.org/10.1177/1745691620917333.

(4) Yu, Z.; Guindani, M.; Grieco, S. F.; Chen, L.; Holmes, T. C.; Xu, X. Beyond t Test and ANOVA: Applications of Mixed-Effects Models for More Rigorous Statistical Analysis in Neuroscience Research. *Neuron* **2022**, *110* (1), 21–35. https://doi.org/10.1016/j.neuron.2021.10.030.

(5) R Core Team. R: A Language and Environment for Statistical Computing, 2023. https://www.R-project.org.

(6) Brooks, M., E.; Kristensen, K.; Benthem, K., J. ,van; Magnusson, A.; Berg, C., W.; Nielsen, A.; Skaug, H., J.; Mächler, M.; Bolker, B., M. glmmTMB Balances Speed and Flexibility Among Packages for Zero-Inflated Generalized Linear Mixed Modeling. *The R Journal* **2017**, *9* (2), 378. https://doi.org/10.32614/RJ-2017-066.
