# Supplementary material for: Extinction context is learned by pigeons, not given by the environment
Source: Commun Psychol. 2025 May 24;3:83. doi: 10.1038/s44271-025-00261-2 (PMC12103601; doi:10.1038/s44271-025-00261-2)
Supplement: Supplementary file 3 — Description of additional supplementary video [file 44271_2025_261_MOESM3_ESM.pdf]

Description of additional supplementary data

File name: Supplementary video

Description: The supplementary material includes a short video of one of the experimental sessions, showing a pigeon performing the ABA' paradigm. The video presents an edited session in which the pigeon undergoes the acquisition phase in the blue arm of the arena, engaging with the novel stimuli and learning their new associations during the first few trials. After reaching the acquisition criterion, the pigeon enters a one-minute transition period, during which it drinks water and searches for the new interaction screen, leading into the extinction phase.

In this phase, both the environmental context (now the red arm) and the local context (a yellow background displayed only during the initiation screen) are changed. Finally, the pigeon proceeds to the renewal phase, where the local context changes again—the initiation screen now appears white—while the environmental context remains the same (still the red arm), thus realizing the competing ABA' sequence.
