# Supplementary material for: Extinction context is learned by pigeons, not given by the environment
Source: Commun Psychol. 2025 May 24;3:83. doi: 10.1038/s44271-025-00261-2 (PMC12103601; doi:10.1038/s44271-025-00261-2)
Supplement: Supplementary file 1 — Peer review file [file 44271_2025_261_MOESM1_ESM.pdf]

nature portfolio

Peer Review File

Extinction context is learned by pigeons, not given by the environment

Corresponding Author: Mr Juan Medina Peschken

This file contains all editorial decision letters in order by version, followed by all author rebuttals in order by version.

Version 0:

Decision Letter:

** Please ensure you delete the link to your author homepage in this e-mail if you wish to forward it to your coauthors **

Dear Mr Medina Peschken,

Thank you for your patience during the peer-review process. Your manuscript titled "Context is Learned, not Given" has now been seen by 2 reviewers, whose comments are appended below. You will see that they find your work of some potential interest. However, they have raised quite substantial concerns that must be addressed. In light of these comments, we cannot accept the manuscript for publication, but would be interested in considering a revised version that fully addresses these serious concerns.

We hope you will find the Reviewers' comments useful as you decide how to proceed. Should additional work allow you to address these criticisms, we would be happy to look at a substantially revised manuscript. If you choose to take up this option, please highlight all changes in the manuscript text file, and provide a detailed point-by-point reply to the reviewers.

Please bear in mind that we will be reluctant to approach the reviewers again in the absence of substantial revisions.

Editorially, we consider it crucial that the central results in the revised manuscript are not confounded by other factors such as difference in stimulus saliency, as pointed out by the reviewers. To this end, please clarify how the current experimental design is not affected by the confound or provide additional empirical data to address the reviewers' concerns. In addition, please conduct an additional experiment where a control condition (AAA) and the low temporal contiguity condition for spatial context are included per Reviewer #1's suggestion.

I am attaching a checklist that details critical reporting requirements for the revised manuscript. Please attend to each item and ensure your manuscript is fully compliant. We are requesting that your manuscript aligns with these requirements as this facilitates the evaluation of your manuscript, reducing delays in re-review and potential future acceptance. If your revised manuscript is not aligned with these requests on major issues, such as those concerning statistics, it may be returned to you for further revisions without re-review. Additional information can be found in our style and formatting guide Communications Psychology formatting guide.

If the revision process takes significantly longer than five months, we will be happy to reconsider your paper at a later date, provided it still presents a significant contribution to the literature at that stage.

We would appreciate it if you could keep us informed about an estimated timescale for resubmission, to facilitate our planning.

We are committed to providing a fair and constructive peer-review process. Please do not hesitate to contact us if you wish to discuss the revision in more detail.

Please use the following link to submit your
- revised manuscript,
- point-by-point response to the referees' comments,
- cover letter (as a separate document),

- the Editorial Policy Checklist (see below),
- the Reporting Summary (see below), and
- the completed Editorial Request Table (attached):

Link Redacted

** This url links to your confidential home page and associated information about manuscripts you may have submitted or be reviewing for us. If you wish to forward this email to co-authors, please delete the link to your homepage first **

Thank you for the opportunity to review your work.

Best regards,

Troby Lui

Troby Lui, PhD
Associate Editor
Communications Psychology

REVIEWER EXPERTISE:
Reviewer #1: associative learning, comparative research
Reviewer #2: associative learning, context, comparative research

REVIEWER REPORTS:

Reviewer #1 (Remarks to the Author):

Review of Peschken, Hahn, Pusch and Rose, “Context is learned, not Given”

This manuscript reports the results of two renewal experiments with pigeons in which conditioned stimuli are first trained in the context of local cue A and global cue X. During extinction sessions the same conditioned stimuli are non-reinforced in the context of local cue B and Global Cue Y. Finally, at test, pigeons receive a test of responding to the conditioned stimuli in in the context of either global cue A and local cue Y, or global cue B and local cue X (letters are my addition for ease of description). The temporal contiguity of the local cues was either high (experiment 1) or low (Experiment 2). The results, at test, revealed that conditioned responding recovered more with the reintroduction of the local contextual cue than the global cue in experiment 1, but that this effect was attenuated, and even partially revered in Experiment 2. These results are interpreted in terms of the idea that context is better defined in terms of something that is learned, through association and contiguity, rather than something that corresponds more to the stimulus properties (such as spatial location).

I think this is an interesting research question, both in terms of theoretical importance, and in terms of its potential application to our understanding of the clinical conditions that are implicated in renewal (e.g. drug-use relapse). However, I do not think that the experimental designs employed in this manuscript permit the authors to draw the conclusions that they would like to make.

My principal concern is conceptual. It is very difficult to see how, with the current experimental design, the learning analysis of “what a context is”, could be falsified. The global and local conditions of experiment 1 (for example) differ in many ways; temporal contiguity is emphasised, and that is appropriate, because temporal contiguity is an important variable in the acquisition of associative learning. However, so too are many other variables, such as stimulus salience. For example, in the absence of evidence to the contrary, it is possible that the global context was more salient than the local context. This being the case then we might wish to conclude that associative learning was NOT the more important variable determining context because we would expect a change to a more salient stimulus would impact renewal, but it didn’t. Put another way, it is incumbent upon the experimenter to show that the properties of the spatial/global manipulation are those which DO NOT affect associative learning, but that the properties of the contiguity/local manipulations DO affect associative learning. I am not convinced that the current experiments permit this logical conclusion.

There are two additional, substantial, experimental design issues that meant, for me, it was difficult to be fully clear about the interpretation of these experiments:

(1) No control condition is included to test for the presence of renewal. Typically, renewal would be assessed in an ABA design by comparing performance in this condition with another control condition (e.g. one which does not undergo any contextual change during extinction, thus: AAA training for example). Without these controls it is difficult to know if recovery of responding at test is due to actual contextual dependency or something else (e.g. temporal confounds, recovery from fatigue, attentional re-engagement).

(2) The role of temporal contiguity (and thus the role of associative learning) is demonstrated by increasing the interval between the local context and the CS in Experiment 2. However, it feels like only half of an experiment was done here.

Surely we need an equivalent demonstration of NO impact of a spatial manipulation too (a spatial– condition)? Without this, we do not know if recovery wouldn't also have been disrupted by changes in the spatial/physical characteristics of the context (and surely it would, right?)

Additionally, we are told on page 5 (paragraph starting on line 145) that the context was constant during each acquisition session but that across sessions the spatial context was balanced, with each of the touchscreen locations being used in only one acquisition. However, the local context was white during all sessions. This appears to imply a significant experimental confound - the local context was repeatedly used during the experiment for each animal, but the global context was variable.

Finally, I like the inclusion of the familiar S+ and S- to keep motivation/performance ongoing throughout the experiment, but it does introduce the problem of establishing the touchscreens as a source of information that is constant/relevant, the global contexts do not benefit from this.

Some clarifications that could be made to help the reader:

What was the background colour of the touch screens when the local context was not presented in Experiment 2?

I assume that pigeons are placed into each context at the start of a session and then removed at the end, rather than being permitted to freely move between chambers, but (unless I missed it) this is not mentioned.

At test, each context was tested 4 times. What was the inter-test interval? What happened to the birds during these intervals, were they taken out of the box?

Without some further definitions and justifications, the equation at the top of page 8 is not very clear.

Mark Haselgrove (Signed)

Reviewer #2 (Remarks to the Author):

Ms COMMSPSYCHOL-24-0756

In their ms, "Context is Learned, not Given," Peschken and his colleagues address what the context is in an experimental situation involving extinction and renewal of previously acquired behavior. The authors report that both high spatial and temporal contiguity enhance the modulating properties of potential contextual cues. Their findings suggest that experimenters cannot assume which cues or stimuli will serve as context. Context, in the sense of cues that can modulate a learned association, depends on the organism's learning situation, and it is also part of the learning experience.

This is a very interesting analysis of context, with direct implications for both basic and applied research. The experiments are well-designed and well-conducted, and the results are clear and carefully analyzed.

The study was preregistered, and the preregistration was followed except for the statistical techniques used to analyze the data. The statistics that were conducted are more appropriate and sophisticated than those initially proposed, and the study and its conclusions certainly gain from this departure.

I recommend accepting the manuscript, but the following issues should be addressed and discussed in a revision:

1) In Experiment 2, the similarity between the extinction local context and the renewal local context is greater than in Experiment 1. Could this be the reason for the absence of renewal in Experiment 2?

2) The local cues in Experiment 1 (specifically, the background color of the screen where the stimuli pairs are displayed) are clearly more salient; they cannot be missed, given that they surround the target stimuli. Is higher salience just the consequence of high spatial and temporal contiguity? Some discussion of the relationship of these concepts would be welcome.

3) The local context exerts a larger influence in Experiment 1 than Experiment 2, and prompts more renewal responses. But a return to the acquisition spatial context (by the way, I'm not sure whether "spatial" is the best term here, given that the local context also has spatial properties; perhaps "environment"?) also engenders renewal responses (in both Experiments 1 and 2). Could the authors include some discussion about the relative ability of the two types of contexts to elicit renewal? Right now, they seem to ignore that the spatial context also works to elicit renewal.

4) I understand (and I agree) that the difference between the local contexts in Experiments 1 and 2 involves spatial and temporal contiguity. But this difference can also be described as a difference between processing simultaneous and sequential information. Pigeons seem to come more easily under the control of simultaneously than sequentially presented information (e.g., O'Donoghue et al., 2022), perhaps because processing of sequential information is more taxing on working memory. It could be that the pigeons are ignoring the background color of the initiation screen in Experiment 2, and that is the reason for the absence of renewal. Do the authors have some data that indicate that manipulation of the initiation screen can lead to differences in the pigeons' behavior?

5) The data reported are consistent with prior research suggesting that pigeons are inclined to process information within a relatively restricted attentional window, and that they may be more sensitive to local than to global stimulus features (e.g., Cavoto & Cook, 2001; Goto et al., 2004). This prior research should be acknowledged.

References:

Cavoto, K. K., & Cook, R. G. (2001). Cognitive precedence for local information in hierarchical stimulus processing by pigeons. Journal of Experimental Psychology: Animal Behavior Processes, 27(1), 3–16. https://doi.org/10.1037/0097-7403.27.1.3

Goto, K., Wills, A. J., & Lea, S. E. (2004). Global-feature classification can be acquired more rapidly than local-feature classification in both humans and pigeons. Animal Cognition, 7(2), 109–113. https://doi.org/10.1007/s10071-003-0193-8

O'Donoghue, E. M., Castro, L., & Wasserman, E. A. (2022). Hierarchical and configural control in conditional discrimination learning. Journal of Experimental Psychology: Animal Learning and Cognition, 48(4), 370–382. https://doi.org/10.1037/xan0000342

EDITORIAL POLICIES

We ask that you ensure your manuscript complies with our editorial policies and reporting requirements.

To that end, we require revised manuscripts to be accompanied by two completed items: a reporting summary that collects information on study design and procedure, and an editorial policy checklist that verifies compliance with all required editorial policies

- Nature Research Reporting Summary
- Editorial Policy Checklist

All points on the policy checklist must be addressed. Your revised manuscript can only be sent back to the referees if these checklists are completed and uploaded with the revision.

Notes: If you have submitted a Stage 1 Registered Report, Review, Primer, Comment, or Perspective you do not need to submit these forms. If you have already submitted these forms, you may disregard this request.

* TRANSPARENT PEER REVIEW: Communications Psychology uses a transparent peer review system. This means that we publish the editorial decision letters including Reviewers' comments to the authors and the author rebuttal letters online as a supplementary peer review file. However, on author request, confidential information and data can be removed from the published reviewer reports and rebuttal letters prior to publication. If your manuscript has been previously reviewed at another journal, those Reviewers' comments would not form part of the published peer review file.

** Visit Nature Research's author and referees' website at www.nature.com/authors for information about policies, services and author benefits**

Communications Psychology is committed to improving transparency in authorship. As part of our efforts in this direction, we are now requesting that all authors identified as ‘corresponding author’ create and link their Open Researcher and Contributor Identifier (ORCID) with their account on the Manuscript Tracking System prior to acceptance. ORCID helps the scientific community achieve unambiguous attribution of all scholarly contributions. You can create and link your ORCID from the home page of the Manuscript Tracking System by clicking on ‘Modify my Springer Nature account’ and following the instructions in the link below. Please also inform all co-authors that they can add their ORCIDs to their accounts and that they must do so prior to acceptance.
https://www.springernature.com/gp/researchers/orcid/orcid-for-nature-research

For more information please visit http://www.springernature.com/orcid

If you experience problems in linking your ORCID, please contact the Platform Support Helpdesk.

Version 1:

Decision Letter:

** Please ensure you delete the link to your author homepage in this e-mail if you wish to forward it to your coauthors **

Dear Mr Medina Peschken,

Your manuscript titled "Context is Learned, not Given" has now been seen by our reviewers, whose comments appear below. In light of their advice I am delighted to say that we are happy, in principle, to publish a suitably revised version in Communications Psychology.

We therefore invite you to revise your paper one last time to address the remaining concerns of our reviewers and a list of editorial requests. At the same time we ask that you edit your manuscript to comply with our format requirements and to maximise the accessibility and therefore the impact of your work.

EDITORIAL REQUESTS:

Please make sure that your preregistration reporting (currently under "Planned study protocol") complies with our preregistration policy. Authors must disclose all deviations from the preregistered protocol and explain the rationale for deviation (e.g., flaw, feasibility, suboptimality). In cases of deviation from the preregistered analysis plan for reasons other than fundamental flaw or feasibility, the originally planned analyses must also be reported. You can find our full policy on preregistration here: https://www.nature.com/commspsychol/submit/preregistration

Please review our specific editorial comments and requests regarding your manuscript in the attached "Editorial Requests Table". Please outline your response to each request in the right hand column. Please upload the completed table with your manuscript files as a Related Manuscript file.

If you have any questions or concerns about any of our requests, please do not hesitate to contact me.

SUBMISSION INFORMATION:

In order to accept your paper, we require the files listed here https://www.nature.com/documents/commsj-file-checklist.pdf .

OPEN ACCESS:

Communications Psychology is a fully open access journal. Articles are made freely accessible on publication. For further information about article processing charges, open access funding, and advice and support from Nature Research, please visit https://www.nature.com/commspsychol/open-access

At acceptance, you will be provided with instructions for completing the open access licence agreement on behalf of all authors. This grants us the necessary permissions to publish your paper. Additionally, you will be asked to declare that all required third party permissions have been obtained, and to provide billing information in order to pay the article-processing charge (APC).

* TRANSPARENT PEER REVIEW: Communications Psychology uses a transparent peer review system. On author request, confidential information and data can be removed from the published reviewer reports and rebuttal letters prior to publication. If you are concerned about the release of confidential data, please let us know specifically what information you would like to have removed. Please note that we cannot incorporate redactions for any other reasons.

* CODE AVAILABILITY: All Communications Psychology manuscripts must include a section titled "Code Availability" at the end of the methods section. We require that the custom analysis code supporting your conclusions is made available in a publicly accessible repository at this stage; please choose a repository that generates a digital object identifier (DOI) for the code; the link to the repository and the DOI must be included in the Code Availability statement. Publication as Supplementary Information will not suffice.

* DATA AVAILABILITY:
All Communications Psychology manuscripts must include a section titled "Data Availability" at the end of the Methods section. More information on this policy, is available in the Editorial Requests Table and at http://www.nature.com/authors/policies/data/data-availability-statements-data-citations.pdf.

Please use the following link to submit the above items:
Link Redacted
** This url links to your confidential home page and associated information about manuscripts you may have submitted or be reviewing for us. If you wish to forward this email to co-authors, please delete the link to your homepage first **

We hope to hear from you within two weeks; please let us know if you need more time.

Best regards,

Troby Lui

Troby Lui, PhD
Associate Editor
Communications Psychology

REVIEWERS' COMMENTS:

Reviewer #2 (Remarks to the Author):

I was Reviewer 2 on the first review round of Peschken et al.'s ms.

Their rebuttal letter is very thorough and clear. All my points (I'd say, all points in general) are carefully considered and addressed. When appropriate, the authors included changes in the ms that make the ms clearer and even more interesting. The addition of the movie is also welcome. I don't have any further concerns, so I highly recommend publication of this study.

This study will be a great contribution to the literature on what the context is and the role of context at many different levels. Congratulations to the authors!

Reviewer #3 (Remarks to the Author):

The manuscript by Peschken and colleagues reports the results of 2 provocative studies aimed at clarifying the nature of context. Using renewal experiments with pigeons, the authors argue that context is better defined in terms of something that is learned, through association and contiguity, rather than something that corresponds more to the stimulus properties (such as spatial location).

Reading through both the manuscript and the previous reviews, I found the studies elegant and the manuscript well written. I also found the revisions to be thoughtful responses to the criticisms of the 2 initial reviewers (some of which I shared). Importantly, I agree with the authors' assertion that the inclusion of the salience data (which they show in response to reviewer 1) would overburden the manuscript and reduce clarity overall. These data are better left for an independent follow-up paper.

For these reasons, I believe the manuscript should be accepted.

** Visit Nature Research's author and referees' website at www.nature.com/authors for information about policies, services and author benefits**

Open Access This Peer Review File is licensed under a Creative Commons Attribution 4.0 International License, which permits use, sharing, adaptation, distribution and reproduction in any medium or format, as long as you give appropriate credit to the original author(s) and the source, provide a link to the Creative Commons license, and indicate if changes were made.
In cases where reviewers are anonymous, credit should be given to 'Anonymous Referee' and the source.
The images or other third party material in this Peer Review File are included in the article's Creative Commons license, unless indicated otherwise in a credit line to the material. If material is not included in the article's Creative Commons license and your intended use is not permitted by statutory regulation or exceeds the permitted use, you will need to obtain permission directly from the copyright holder.
To view a copy of this license, visit https://creativecommons.org/licenses/by/4.0/

Context is Learned, not Given / COMMSPSYCHOL-24-0756

Point-by-point response to the reviewers' comments

- Reviewer's comments are shown in *Italic.*
- Our response to each point is located below each comment.
- Reference to specific lines in the original manuscript are denoted O.L. and in the revised manuscript as R.L.

General note:

We thank both reviewers and the editor for their time and thoughtful feedback throughout this review process. We are pleased to see that our work has sparked interest and meaningful discussions, particularly regarding its broader implications for the field and future research. One of our main goals in defining context was to provoke critical thinking and encourage the field to re-evaluate classical definitions and implementations. We are pleased to see this engagement reflected in the review process.

Reviewer # 1

This manuscript reports the results of two renewal experiments with pigeons in which conditioned stimuli are first trained in the context of local cue A and global cue X. During extinction sessions the same conditioned stimuli are non-reinforced in the context of local cue B and Global Cue Y. Finally, at test, pigeons receive a test of responding to the conditioned stimuli in in the context of either global cue A and local cue Y, or global cue B and local cue X (letters are my addition for ease of description). The temporal contiguity of the local cues was either high (experiment 1) or low (Experiment 2). The results, at test, revealed that conditioned responding recovered more with the reintroduction of the local contextual cue than the global cue in experiment 1, but that this effect was attenuated, and even partially revered in Experiment 2. These results are interpreted in terms of the idea that context is better defined in terms of something that is learned, through association and contiguity, rather than something that corresponds more to the stimulus properties (such as spatial location).

We appreciate the thorough understanding of our study. The use of the term "global" aligns with Reviewer 2's comment regarding the need for a more accessible description of our spatial condition to facilitate the understanding of our work. We have decided to adopt the suggestion of using "environmental" and will refer to the spatial context as the "environmental context" throughout the revised manuscript.

I think this is an interesting research question, both in terms of theoretical importance, and in terms of its potential application to our understanding of the clinical conditions that are implicated in renewal (e.g. drug-use relapse). However, I do not think that the experimental designs employed in this manuscript permit the authors to draw the conclusions that they would like to make.

We agree with your evaluation regarding the relevance of the research question. However, we are confident that, following the reviewers' constructive feedback, we have clarified our design, added key information to the Methods section, and expanded the Discussion to provide sufficient support for our conclusions.

My principal concern is conceptual. It is very difficult to see how, with the current experimental design, the learning analysis of "what a context is", could be falsified. The global and local conditions of experiment 1 (for example) differ in many ways; temporal contiguity is emphasised, and that is appropriate, because temporal contiguity is an important variable in the acquisition of associative learning. However, so too are many other variables, such as

stimulus salience. For example, in the absence of evidence to the contrary, it is possible that the global context was more salient than the local context. This being the case then we might wish to conclude that associative learning was NOT the more important variable determining context because we would expect a change to a more salient stimulus would impact renewal, but it didn't. Put another way, it is incumbent upon the experimenter to show that the properties of the spatial/global manipulation are those which DO NOT affect associative learning, but that the properties of the contiguity/local manipulations DO affect associative learning. I am not convinced that the current experiments permit this logical conclusion.

You note that the two conditions we compared (environmental and local context) may differ in several ways, one of which could be saliency. However, we believe this misses one important point. Common definitions of context suggest that environment should be the ideal stimulus, whereas the local stimulus, no matter how well constructed, would not typically meet this definition. Yet, our results show that local best predicts renewal. Only after demonstrating this, do we proceed in experiment 2 to show that reducing its associative strength by lowering contiguity also reduces its ability of to generate renewal.

However, we understand the concern regarding the role of saliency and its potential impact on the difficulty of falsifying the learning analysis of "what a context is." To address this, we will explicitly outline our line of reasoning using saliency as an example.

There is no doubt that saliency is a key factor in associative learning. As such, it must play a critical role if context is, as we argue, indeed learned. However, if our conclusions were incorrect and context was best understood as the environment of the animal, the situation would be very different. Context would not (fully) obey learning rules, and the environment would, by definition, be the optimal context stimulus. In other words, experimental control of saliency becomes critical under the premise that context is learned, since saliency would be a predictor of associative strength. The alternative perspective, which sees context as the environment, would not predict a direct relationship between strength of renewal and saliency, as the animals would only need to detect environmental changes. However, this is not what we found: In our experiments, pigeons did show renewal with the environmental context—therefore, it was salient enough to be detected as context—yet it was weaker than the local context.

In an additional experiment (mentioned in our preregistration), we indirectly confirmed that the environmental stimuli obey the influence of saliency. We chose not to include this data here but to publish it later to avoid overloading the manuscript, as there are many differences in the protocol that would make the data difficult to integrate. Briefly, the other study included less salient environmental stimuli. Comparing renewal between this less salient environmental context and the current, more salient one, your prediction is precisely met: Greater saliency leads to stronger renewal (see supplementary Figure). This observation strengthens our interpretation that any context is learned, including the spatial information present in the environmental context, and its effectiveness is shaped by learning parameters (here, saliency) rather than given by inherent stimulus properties.

We firmly believe that differences between experimental procedures would make the manuscript harder to read and, therefore, less suitable for broad readership. We reiterate that we agree with your observation on the relevance of exploring stimulus saliency under the premise of associative learning. In fact, an additional study that precisely controls stimulus saliency, with an adjusted psychophysical curve, is already underway. However, this line of investigation requires significantly more time (we expect 6–9 months) and effort, and it falls beyond the scope of the current manuscript.

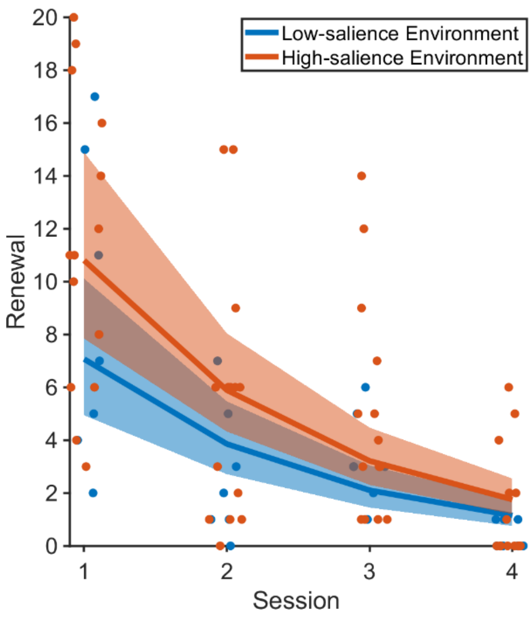

Supplementary Figure: The environmental context in this alternative experiment (shown in blue and labeled "Low-salience Environment") was based solely on the bird's location within the arena. No specific illumination was provided to any arm, resulting in a uniformly white arena. Additionally, the screens at the end of each arm were used as a third distinctive contextual condition. These screens contingently changed their background color across phases and did not follow the pattern employed in the current study. The "High-salience Environment" (shown in red) corresponds to the environmental context described in the present study. As depicted in the figure, higher salience in the environmental context produced stronger renewal across all testing sessions. Significant differences were assessed using a Poisson model that examined the number of renewal responses across sessions in both experiments (β = 0.424, t = 3.790, $p < 0.001$).

There are two additional, substantial, experimental design issues that meant, for me, it was difficult to be fully clear about the interpretation of these experiments:

(1) No control condition is included to test for the presence of renewal. Typically, renewal would be assessed in an ABA design by comparing performance in this condition with another control condition (e.g. one which does not undergo any contextual change during extinction, thus: AAA training for example). Without these controls it is difficult to know if recovery of responding at test is due to actual contextual dependency or something else (e.g. temporal confounds, recovery from fatigue, attentional re-engagement).

We thank you for suggesting including an AAA control and for mentioning that this is common in the literature. It is indeed a critical control in many classical ABA procedures since these are often run across several sessions / days. As pointed out, in that framework, confounds such as reduced fatigue could cause renewal. However, our design is run within a single session with only one minute interval between the experimental phases. This implies greater similarity between the experimental phases and makes accounts such as reduction of fatigue unlikely between extinction and renewal. If the same approach were used in an AAA fashion, we predict there would be no reason for the animal to reengage with the extinguished stimuli and to spontaneously show recovery. In principle, if the goal is to control potential explanations for renewal, avoiding any contextual change would fundamentally violate the very definition of renewal.

The within-session protocol is well established in operant chambers, it was used to investigate extinction behaviorally (1) and it was used to reveal neural correlates of extinction and, importantly, of context (2) clearly demonstrating that it covers extinction learning as expected.

We would like to remark that some important controls are already implemented through the inclusion of familiar stimuli. Potential confounds such as fatigue recovery or satiation are controlled because the animal never changes its engagement level in the task, continuing to be reinforced by the familiar stimuli alone. This, in turn, also controls for possible attentional re-engagement, as the pigeon remains consistently involved in the experiment. We believe that clarification of our protocol in the methods and a discussion of the implications of a within-session procedure should remedy your concerns. This can be found in R.L 127-129: “Conditioned responses to the familiar stimuli (S+) were always rewarded, allowing us to manage satiety levels and potential fatigue during the session.” And in R.L. 175-177: “During renewal, the novel stimuli remained without feedback exactly

as in extinction. Furthermore, renewal was induced just one minute later. These two factors allow us to control for potential recovery effects, such as reinstatement and spontaneous recovery, ensuring that the observed responses are a result of the renewal effect.”

1. Packheiser, J., Güntürkün, O. & Pusch, R. Renewal of extinguished behavior in pigeons (Columba livia) does not require memory consolidation of acquisition or extinction in a free-operant appetitive conditioning paradigm. Behavioural Brain Research 370, 111947 (2019). https://doi.org/10.1016/j.bbr.2019.111947

2. Packheiser, J., Donoso, J. R., Cheng, S., Güntürkün, O. & Pusch, R. Trial-by-trial dynamics of reward prediction error-associated signals during extinction learning and renewal. Progress in Neurobiology 197, 101901 (2021). https://doi.org/10.1016/j.pneurobio.2020.101901

(2) The role of temporal contiguity (and thus the role of associative learning) is demonstrated by increasing the interval between the local context and the CS in Experiment 2. However, it feels like only half of an experiment was done here. Surely, we need an equivalent demonstration of NO impact of a spatial manipulation too (a spatial– condition)? Without this, we do not know if recovery wouldn’t also have been disrupted by changes in the spatial/physical characteristics of the context (and surely it would, right?)

We appreciate this suggestion, nevertheless we believe that some of the concerns regarding a spatial manipulation are, in part, due to a lack of clarity in our manuscript, potentially accompanied by misunderstandings of the protocol (this refers to a later comment about the movement of the animals between boxes).

Our pigeons move freely through the open and continuous space in the arena. This implies that the differentiation of the environment is based on the animal's movement and the conditions it learns from exploring the space in continuous time. We have thoroughly reviewed the Methods section to make this clear and understandable. Additionally, the inclusion of a video showing a pigeon in the paradigm (The video has been uploaded to the "Manuscript Tracking System" platform and should be available to reviewers. To ensure accessibility, we also provide a secondary link: https://ruhr-uni-bochum.sciebo.de/s/0wbNt2EOMe9e8f9) should further increase the clarity of our novel experimental design.

In terms of a potential manipulation of spatial contiguity or a “spatial“ condition, this would indeed be an interesting control to add. However, as mentioned, the pigeon moves freely and uninterrupted through space in our setup. We do not see how we could manipulate the contiguity (spatio-temporal simultaneity) of this space. In fact, it may be, in principle, impossible to dissociate the temporal from the spatial surroundings of the animal during task performance. Performance in the paradigm will implicitly always occur while the animal occupies a certain space. However, we remain open to specific suggestions that could guide us in addressing this.

Additionally, as mentioned previously, we propose that context representation is mediated by properties of learning, and that any stimulus presented as a potential context would be governed by the same principles. This would also apply to the learning of environmental information as context. Consequently, we would expect that, if possible, a manipulation of spatial contiguity would also affect the recovery of conditioned responses. This was already partially demonstrated and discussed in the saliency data above.

Additionally, we are told on page 5 (paragraph starting on line 145) that the context was constant during each acquisition session but that across sessions the spatial context was balanced, with each of the touchscreen locations being used in only one acquisition. However, the local context was white during all sessions. This appears to imply a significant experimental

confound - the local context was repeatedly used during the experiment for each animal, but the global context was variable.

This could stem from a poor choice of words on our part in the text. To clarify, we have revised the line in question, now R.L. 151 express: "The physical array of the arena was constant during each acquisition." This control was intentionally designed to ensure both contexts were treated equally. The pigeons underwent training in the arena and visited each location countless times, ensuring that no spatial bias was introduced. Likewise, all training sessions were conducted with a white background, signaling a stable feature for the pigeons. Had we treated the local context differently, we would have introduced a potential confound that could artificially enhance the renewal effect. Specifically, novelty in the acquisition local context could have influenced context learning and subsequent renewal, making it unclear whether the observed results were due to differences in contiguity or novelty.

Finally, I like the inclusion of the familiar S+ and S- to keep motivation/performance ongoing throughout the experiment, but it does introduce the problem of establishing the touchscreens as a source of information that is constant/relevant, the global contexts do not benefit from this.

We agree that the inclusion of the familiar stimulus helps maintain high motivation throughout the experiment. However, it is not possible for the relevance of this stimulus to be associated exclusively with local information and not with the environmental information. Again, anything the animal does or perceives must happen in the context of its environment. The stimuli get presented as much on a touchscreen as they do in the environment of a specific spatial location that the animal actively walked into and that has a specific-colored illumination that is always visually accessible to the animal.

Some clarifications that could be made to help the reader:

What was the background colour of the touch screens when the local context was not presented in Experiment 2?

O.L. 191-193 addressed this point, this is now further clarified in R.L. 204 with “The touchscreen remained white”.

I assume that pigeons are placed into each context at the start of a session and then removed at the end, rather than being permitted to freely move between chambers, but (unless I missed it) this is not mentioned.

O.L 151-152 / 162-163, address this comment. Consequently, following those lines, we have now included the phrases: “Here, the bird roamed freely, looking for the next active touchscreen to continue the task in the extinction phase” and “During this ITI, the animal moves through the arena in search of the final active screen.” which should clarify this point.

At test, each context was tested 4 times. What was the inter-test interval? What happened to the birds during these intervals, were they taken out of the box?

O.L. 200-204 now R.L 211-216 explained the procedure and address this comment. We would like to reiterate It here: In short, all animals were randomly assigned to experience either the local or environmental context in their first session. They then alternated between contexts in each subsequent session, with one session per day on consecutive days, and each context being tested four times.

Without some further definitions and justifications, the equation at the top of page 8 is not very clear.

It is possible that, due to an unfortunate page break between the end of page 7 and the beginning of page 8, it may be difficult to associate the information describing the equation. O.L. 217-221 explicitly refer to the equation, now we expanded it in R.L 235-238.

Reviewer # 2

In their ms, “Context is Learned, not Given,” Peschken and his colleagues address what the context is in an experimental situation involving extinction and renewal of previously acquired behavior. The authors report that both high spatial and temporal contiguity enhance the modulating properties of potential contextual cues. Their findings suggest that experimenters cannot assume which cues or stimuli will serve as context. Context, in the sense of cues that can modulate a learned association, depends on the organism’s learning situation, and it is also part of the learning experience.

This is a very interesting analysis of context, with direct implications for both basic and applied research. The experiments are well-designed and well-conducted, and the results are clear and carefully analyzed.

The study was preregistered, and the preregistration was followed except for the statistical techniques used to analyze the data. The statistics that were conducted are more appropriate and sophisticated than those initially proposed, and the study and its conclusions certainly gain from this departure.

We appreciate the thoughtful assessment and fully agree with the relevance of our study's implications. Despite the extensive literature on context in extinction learning, the lack of clarity about what context truly is, remains critical. We are also grateful for the acknowledgment of the preregistration process and agree that deviating from the original statistical analysis strengthened the manuscript.

I recommend accepting the manuscript, but the following issues should be addressed and discussed in a revision:

1) In Experiment 2, the similarity between the extinction local context and the renewal local context is greater than in Experiment 1. Could this be the reason for the absence of renewal in Experiment 2?

The observation regarding similarity, and the subsequent points on simultaneous versus sequential processing and pigeons' sensitivity to local rather than global features fundamentally address potential explanations for our results. These are a great inclusion to the study and are now discussed in R.L. 361-384. However, we want to emphasize that there was no difference in the physical properties of the local stimulus between the two experiments—the only change was in contiguity. If this increased the similarity between the local context during extinction and renewal, we could then frame it as greater similarity in the temporal domain. In other words, this is essentially restating our contiguity argument using different terms.

2) The local cues in Experiment 1 (specifically, the background color of the screen where the stimuli pairs are displayed) are clearly more salient; they cannot be missed, given that they surround the target stimuli. Is higher salience just the consequence of high spatial and temporal contiguity? Some discussion of the relationship of these concepts would be welcome.

This is a valid observation and aligns with Reviewer 1's comment on saliency (please also refer to our discussion of the comment of Reviewer 1). Our response to that point offers arguments that may address your concern. However, while the revised Discussion now presents the issue of saliency in a more integrated manner, here we specifically address

the inevitability of the local context and the potential omission of the environmental context.

We believe it is highly unlikely, for the environmental information to be omitted or missed entirely in our paradigm. First, the open and continuous nature of the space in our arena requires voluntary movement of the birds between different sections. Second, the visual system of pigeons has been extensively studied, and it is well-established that they can attend to multiple spatial areas simultaneously, even through independent neural pathways (3). The arena setup provides distinct illumination patterns and stable landmarks that inform the pigeons of their location. Consequently, pigeons not only have access to varying lighting conditions that result in different wall colors surrounding the touchscreen (which in turn surrounds the stimuli) but also simultaneously receive landmark information through their lateral vision. We included a video of the animals performing in the paradigm to illustrate just how drastic the different locations in the arena look (The video has been uploaded to the "Manuscript Tracking System" platform and should be available to reviewers. To ensure accessibility, we also provide a secondary link: https://ruhr-uni-bochum.sciebo.de/s/0wbNt2EOMe9e8f9).

Finally, the question of whether salience is merely a consequence of high contiguity is highly relevant, we have expanded its discussion as requested. We believe that this comment has strengthened our argument of context as a product of learning, and we thank you for it.

3. Clark, W. & Colombo, M. Seeing the Forest for the Trees, and the Ground Below My Beak: Global and Local Processing in the Pigeon’s Visual System. Front. Psychol. 13, 888528 (2022). https://doi.org/10.3389/fpsyg.2022.888528

3) The local context exerts a larger influence in Experiment 1 than Experiment 2 and prompts more renewal responses. But a return to the acquisition spatial context (by the way, I’m not sure whether “spatial” is the best term here, given that the local context also has spatial properties; perhaps “environment”?) also engenders renewal responses (in both Experiments 1 and 2). Could the authors include some discussion about the relative ability of the two types of contexts to elicit renewal? Right now, they seem to ignore that the spatial context also works to elicit renewal.

First, we appreciate your suggestion to change the term 'spatial' to 'environmental.' We believe this adjustment provides important clarification for readers and aligns with the use of the term 'global' by Reviewer 1. Accordingly, we have decided to refer to the spatial context as the "environmental context" throughout the revised manuscript.

Second, in O.L 258 / 287-288 / 295-297 / 313-315, we directly address the capacity of the environmental context to elicit renewal. In accordance with your request, in R.L 339-348 we have now expanded the discussion to include the ability of the environmental context to produce renewal and expand on how our proposed mechanism may explain the many different, and successful, implementations of environmental stimuli in the literature.

4) I understand (and I agree) that the difference between the local contexts in Experiments 1 and 2 involves spatial and temporal contiguity. But this difference can also be described as a difference between processing simultaneous and sequential information. Pigeons seem to come more easily under the control of simultaneously than sequentially presented information (e.g., O’Donoghue et al., 2022), perhaps because processing of sequential information is more taxing on working memory. It could be that the pigeons are ignoring the background color of the initiation screen in Experiment 2, and that is the reason for the absence of renewal. Do the authors have some data that indicate that manipulation of the initiation screen can lead to differences in the pigeons’ behavior?

This is an interesting point that we have now incorporated into the discussion, specifically in R.L 361-372.

5) The data reported are consistent with prior research suggesting that pigeons are inclined to process information within a relatively restricted attentional window, and that they may be more sensitive to local than to global stimulus features (e.g., Cavoto & Cook, 2001; Goto et al., 2004). This prior research should be acknowledged.

We agree with this comment and appreciate the suggestion of relevant literature. The two studies have been added to the revised manuscript and incorporated into the discussion under R.L 373-384.
